# Nanomaterials Designed for Antiviral Drug Delivery Transport across Biological Barriers

**DOI:** 10.3390/pharmaceutics12020171

**Published:** 2020-02-18

**Authors:** Florina-Daniela Cojocaru, Doru Botezat, Ioannis Gardikiotis, Cristina-Mariana Uritu, Gianina Dodi, Laura Trandafir, Ciprian Rezus, Elena Rezus, Bogdan-Ionel Tamba, Cosmin-Teodor Mihai

**Affiliations:** 1 Advanced Centre for Research-Development in Experimental Medicine, Grigore T. Popa University of Medicine and Pharmacy of Iasi, 700115 Iasi, Romania; florina.cojocaru@umfiasi.ro (F.-D.C.); doru.botezat@umfiasi.ro (D.B.); ioannis.gardikiotis@umfiasi.ro (I.G.); cristina-mariana.uritu@umfiasi.ro (C.-M.U.); bogdan.tamba@umfiasi.ro (B.-I.T.); cosmin-teodor.mihai@umfiasi.ro (C.-T.M.); 2 Pediatric Department, Grigore T. Popa University of Medicine and Pharmacy of Iasi, 700115 Iasi, Romania; laura.trandafir@umfiasi.ro; 3 Department of Internal Medicine, Grigore T. Popa University of Medicine and Pharmacy, 700115 Iasi, Romania; ciprian.rezus@umfiasi.ro; 4 Department of Rheumatology and Physiotherapy, Grigore T. Popa University of Medicine and Pharmacy, 700115 Iasi, Romania; elena.rezus@umfiasi.ro; 5 NIRDBS—Institute of Biological Research Iasi, Department of Experimental and Applied Biology, 700107 Iasi, Romania

**Keywords:** nanomaterials, antivirals, drug delivery, biological barriers

## Abstract

Viral infections are a major global health problem, representing a significant cause of mortality with an unfavorable continuously amplified socio-economic impact. The increased drug resistance and constant viral replication have been the trigger for important studies regarding the use of nanotechnology in antiviral therapies. Nanomaterials offer unique physico-chemical properties that have linked benefits for drug delivery as ideal tools for viral treatment. Currently, different types of nanomaterials namely nanoparticles, liposomes, nanospheres, nanogels, nanosuspensions and nanoemulsions were studied either in vitro or in vivo for drug delivery of antiviral agents with prospects to be translated in clinical practice. This review highlights the drug delivery nanosystems incorporating the major antiviral classes and their transport across specific barriers at cellular and intracellular level. Important reflections on nanomedicines currently approved or undergoing investigations for the treatment of viral infections are also discussed. Finally, the authors present an overview on the requirements for the design of antiviral nanotherapeutics.

## 1. Social Impact and Economic Burden of Viral Infectious Diseases

Today, we are living through a so-called fourth great transitional period, after the other three waves of epidemiological transitions, namely early agrarian-based settlements, early Eurasian civilizations and European expansionism (more details can be found in McMichael’s description [1]). The current configuration and variety of infectious diseases closely followed the combined evolutions in demography, environment, technology, social change and behaviours. Medicine itself has created new opportunities for microbes either through blood transfusions, organ transplants, the use of hypodermic syringes or the excessive use of antibiotics thus contributing to the induction of iatrogenic effects in some treatments for infections such as hepatitis C, HIV and others [1].

In recent decades, old concerns have been reactivated at both the official and the general public levels regarding infectious diseases as a threat to public health. McMichael [1] analyzed the reflection of this issue in social media and noticed the “emergence and resurgence” of infectious diseases (determined by environmental, sociological and economic changes) and a so-called “public anxiety” set on this topic.

Despite their widespread and increasing transmission, there is still a poor understanding of global economic impact of viral diseases, which makes difficult to evaluate the societal costs and the cost-effectiveness of preventive efforts. The issue of estimating a general impact of viral infections involves several aspects that hinder this approach, namely: the variety of viral infections, the incidence of associated co-morbidities, social and psychological issues having economic repercussions (hidden costs), the variety of treatments (only direct-acting antiviral (DAA) and vaccinations can be used in the evaluation) and the presence of negative externalities [2], meaning that the disease consequences are not limited to their patients infected or potentially but also to the related families who may experience social distress as well. Also, human mobility and long-distance trade have increased; ever-larger cities, often girded with slums, have become highways for microbial traffic; poverty perpetuates vulnerability to infectious disease; and sexual practices, drug injecting, intensified food production and much modern medical technology all create new “audience” for microbial opportunism, and new management issues for public health decision makers [1].

**Figure 1:**
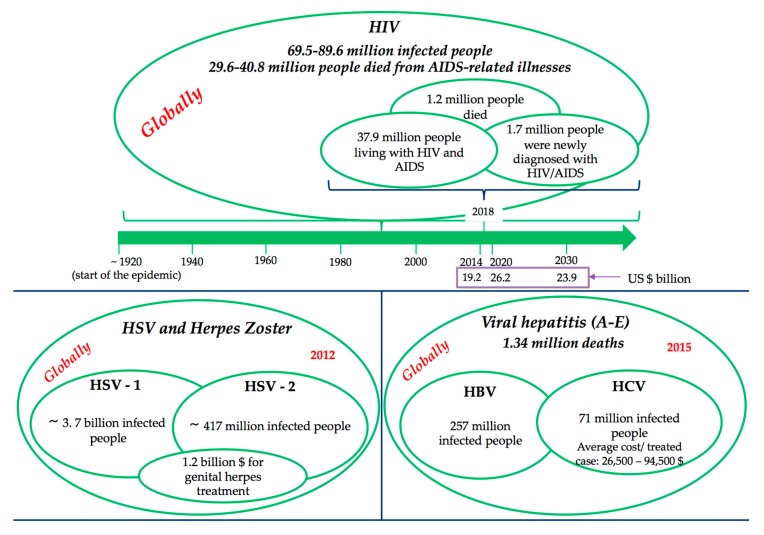
Global impact of viral diseases.

For all these reasons, a global assessment of socio-economic impact seems practically impossible or, if contrary, it could not meet all the criteria to be relevant. Rather, the impact can be split by relevant categories of viral infections, were the literature is more precise but even so, there remain some inconsistencies regarding the unification of the methodologies from the various studies that will ensure the comparability of the data. Figure 1 presents statistical facts related to the most “burden-generator” viral infection diseases [3,4,5,6,7].

HIV/AIDS, considered as one of the major burdens of disease globally, became a chronic disease after the introduction of multiple antiretroviral therapy (ART), and therefore it needs to provide long-term care and support for the ill person, demanding a higher level of treatment costs for the HIV-affected households. Consequently, HIV/AIDS causes depletion of savings and productive assets, and increases the indebtedness of the HIV-affected households [8,9]. Moreover, the higher health care expenditure of the households reduces investment for nutritional food for the family members, investment for farming or business, and the children education. Death during the working age of the patient is a major factor in the economic impact of HIV/AIDS. The household level impact of HIV/AIDS includes direct costs, including medical and non-medical costs, and productivity costs such as loss of labour time, as a result of the morbidity of HIV positive household members, as well as time spent by others caring for them [10]. This evidence suggests that HIV/AIDS places significant economic pressure on households trying to pay for health care costs, and trying to make up for lost income.

The difficulty in accurately quantifying and explaining the morbidity and mortality related to viral hepatitis stems from the fact that hepatitis deaths are caused by five distinct viruses (hepatitis A–E) with different routes of transmission, or from the fact that death occurs decades after infection, and that when people die with hepatitis-related liver cancer and cirrhosis, these deaths are not always linked to the underlying infection.

Although antiviral therapy appears to be expensive (for example, average cost for a treated HCV case ranges from $26,500 to $94,500 [11]), is considered to be also cost effective when compared with other well-accepted medical interventions [12] due to sustained viral response to therapy, the cost savings and quality-of-life improvement and prolongation of life expectancy from the prevention of HCV complications. In the era of new DAAs, the statement “provide treatment to HCV-patients” generates savings compared to not provide it. Low and middle-income countries may consider HCV-treatment as a cost-saving intervention for the health system, not only in a long-term horizon, but in 5–10 years [5]. Economic impact of no treatment is higher than treatment costs itself. But still, the enthusiasm for DAA therapies, however, has been tempered by two major concerns: the price, still very high, of these medications, and, the challenges patients and clinicians face with respect to drug access in many countries.

HSV and herpes zoster (varicella-zosterian virus—VZV reactivation) are some of the most common infections in humans, with no effective treatment available at this time. The impact on the health degree varies from a very small impairment to severe, disabling forms, in most cases limiting methods are applied to local infection and reducing side effects (pain, manifestations dermis etc.). Also, specific psychological issues are related with this disease, such as negative feelings correlated to the condition following diagnosis, in particular if they have acquired the genital form of the disease. Feelings can include depression, fear of rejection, feelings of isolation, fear of being found out, and self-destructive feelings [6].

As mentioned above, all the available reports present crude estimates rather than precise measures of the economic costs of the illness, because most of the costs are calculated directly on interventions or on medical budgetary chapters, without taking into account the societal losses. Moreover, different approaches are discrepant (can be explained, at least in part, with the influence of compliance to treatment and possible under sampling of subpopulations in the data set) [7]. Also, limitations must be placed on the ability to generalize the results beyond the sample. Moreover, not only the cost matters. The costs of an intervention have to be compared with the results of interventions because the effectiveness of treatments and the efficiency can produce societal gains that must be offset by losses [13].

The usual most accurate methodology to estimate a burden (associated with a disease) is the so-called “cost-of-Illness” [10], and measures all the costs of a particular disease, including the direct, indirect, and intangible dimensions. It is widely accepted that estimating the total social cost of a disease is useful in establishing policy decisions [14]. But there are also other methods as the *cross-sectional surveys* of samples of primary and secondary care physicians, analyzing health care resource utilization or approaches based on the analysis of a large administrative data sets, such as values of spending or drugs consuming lists [15]. Other approaches are used to estimate the number of patients seeking medical treatment, the average medical expenditures (as health inputs employed per unit multiplied by number of units) and estimated national costs. These comprehensive studies can often be advantageous in allocating total national expenditures among the major diagnostic categories [16]. However, regardless of the method, such analyzes are not possible otherwise than inside countries (where the impact determined by cultural and social aspects can vary substantially). But even in the absence of global data of this nature, we still can extract from the information presented the relevant issue for the topic of this paper: the current arrangements in the management of viral infections (treatments, prevention and limitations of spread) are costly and less effective, unaffordable in some cases and burdensome for medical systems. For example, according to the current analysis of Globe Newswire Reports and Data [17], the global antiviral drugs market was valued at $49.87 billion in 2018 and is expected to reach $71.48 billion by year 2026. Sales of antivirals increased by approximately 20% each two years. Moreover, thanks to better diagnostics, innovative drugs and new therapeutics, the market is likely to witness even further future growth. However, the list of viral diseases for which antiviral therapies are available is still relatively short [18].

There are several factors that hinder the development of antiviral drugs:

- Dependence of viruses replication on host cell biosynthetic machinery [19], that leads to a limited number of virus-specific metabolic functions can be targeted by antiviral drugs without any damage to the host;
- the viruses’ functions are specific to each virus, preventing the development of a broad-spectrum antivirals fighting against different viruses that cause similar symptoms. Antivirals developed for some viruses (as HSV and HIV) can treat the acute illness, but do not cure the latent infection. This leads to recurrent or chronic diseases that require treatment for longer periods of time [18].

All these limitations prompted the need for a paradigm shift. The great challenge of antiviral therapies is to move on to developing new drug formulas. This involves changing the physico-chemical and bio-pharmaceutical properties of antiviral molecules using new scientific strategies during the preparation or in dosage configuration.

## 2. Viruses: Types, Current Therapy and Observed Drawbacks

Viruses are sub-microscopic intracellular parasitic particles of genetic material contained in a protein coat, totally dependent by host for cell replication, showing both living and non-living characteristics [20].

Living characteristics of the viruses are represented by the high rate of multiplication (only in living host cells) and by the ability to mutate. The non-living characteristics for viruses consist in acellularity (lack of cytoplasm and organelles), the replication only by using host cell’s metabolic machinery and the composition with DNA or RNA [20]. In humans, viral infections are responsible for different diseases as briefly presented in Table 1.

According to International Committee on Taxonomy of Viruses (ICTV) there are 1 phylum, 2 subphylia, six classes, 10 orders, seven suborders, 89 families, 36 subfamilies, 387 genera, 59 subgenera and 2202 species [43]. Currently, viruses are classified based on their type of nucleic acid (DNA, RNA, single-stranded, double-stranded) and their way of replication, known as Baltimore classification [44], divided as seven Baltimore classes:

- I—dsDNA viruses (e.g., adenoviruses, herpesviruses, poxviruses): enter to the host nucleus and are dependent by host cell polymerases to replicate viral genome. The virus may induce the cell to forcefully undergo cell division, which may lead to transformation of the cell and, ultimately, to cancer.
- II—ssDNA viruses (+ strand or “sense”) *DNA* (e.g., parvoviruses), consists of viruses that have a single-stranded DNA genome of the same polarity as the mRNA. Excepting Parvoviruses, most of them have circular genomes and are replicating within nucleus.
- III—dsRNA viruses (e.g., reoviruses): not dependent by host replication polymerases and their replication (monocistronic) is realized into capsid (in cytoplasm).
- IV—(+)ssRNA viruses (+ strand or sense) *RNA* (e.g., picornaviruses, Togaviruses): the RNA can be directly accessed by ribosomes of the host to form proteins, and use a simple reproduction pathway (viruses with polycistronic mRNA) or a more complex transcription pathway (for which subgenomic mRNAs, ribosomal frameshifting, and proteolytic processing of polyproteins may be used).
- V—(−)ssRNA viruses (− strand or antisense) *RNA* (e.g., orthomyxoviruses, rhabdoviruses), that first must be transcribed by viral polymerases (positive-sense) before can be directly accessed by host ribosomes to form proteins.
- VI—ssRNA-RT viruses (+ strand or sense) RNA with DNA intermediate in life-cycle (e.g., retroviruses), which use the reverse transcriptase to convert the positive-sense RNA into DNA. They are using DNA to create the templates and those are spliced into host genome by integrase.
- VII—dsDNA-RT viruses DNA with RNA intermediate in life-cycle (e.g., hepadnaviruses), dsDNA viruses that replicate through a single-stranded RNA intermediate, which use a pregenome RNA as a template and conversion to DNA is done by a viral reverse transcriptase.

There are distinct stages of viral replication (cell entry, uncoating, transcription of viral genome, translation of viral proteins, post-translational modifications and assembly of virion components) and the classes of antiviral agents that can act at each stage, the correspondence between stage of replication and classes of selective inhibitors being described in detail in reference [45] and their pharmacological properties as it follows in the next section.

### Antiviral Agents Used in Nanotechnology

**Figure 2:**
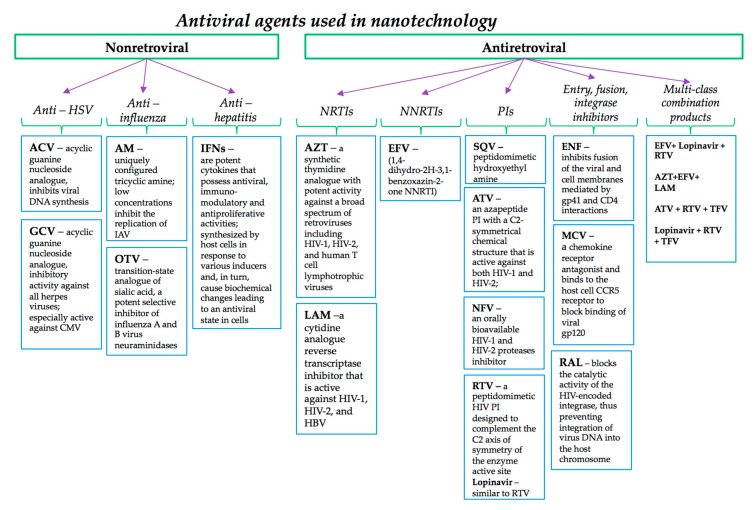
Summary of antiviral agents used in nanotechnology.

According to our knowledge (based on found studies) only the antivirals summarized in Figure 2 were applied in nanomedicine.

The therapy of the large HSV family of double-stranded DNA viruses, widely distributed among humans, includes highly selective and effective antivirals, from which only acyclovir (ACV) and ganciclovir (GCV) were incorporated into nanomaterials; a complete classification of HSV antivirals can be found in described in detail in references [46,47,48,49,50]. Briefly, ACV pharmacology can be explained as follows:

- the ACV selectivity is dependent by interaction with viral HSV thymidine kinase and DNA polymerase, therefore the cellular uptake and first phosphorilation are facilitated by HSV thymidine kinase that presents a high affinity for ACV;
- then, intracellular enzymes convert monophospate to triphosphate ACV and this form of ACV inhibits viral DNA polymerase and, to a much lesser extent, cellular DNA polymerase; ACV triphosphate is also integrated into viral DNA and acts as a chain terminator, as it binds to viral DNA polymerase and determines its irreversible inactivation by a mechanism called suicide inactivation [51,52,53];
- occurrence of resistance to ACV could be acquired by three mechanisms: impaired production of viral thymidine kinase (the most common), altered thymidine kinase substrate specificity (e.g., phosphorylation of thymidine but not acyclovir), or altered viral DNA polymerase (rare). Alterations in viral enzymes are caused by point mutations and base insertions or deletions in the corresponding genes [54].

The mechanism of GCV inhibits viral DNA synthesis [55] as briefly explained below:

- it is monophosphorylated intracellular by viral thymidine kinase during HSV infection and by a viral phosphotransferase encoded by the UL97 gene during CMV infection, while diphosphate and GCV triphosphate forms are produced by cellular enzymes;
- CMV can become resistant to GCV by mutations in the viral phosphotransferase encoded by the UL97 gene and by mutations in viral DNA polymerase [56].

The conventional treatment (prophylaxis or therapy) of an influenza virus infection, as a major public health concern worldwide, is designed to target viral proteins and could be used, either alone or in combination [57]. These include also amantadine and neuraminidase inhibitors (zanamivir and oseltamivir), that have been encapsulated into nanoparticles as specified in Table 2. Potent vacuolar ATPase (V-ATPase) inhibitors, namely diphyllin and bafilomycin, previously shown to have broad-spectrum antiviral activity represent another possibility against influenza virus infection [58,59,60]. Briefly, the antiviral mechanism of amantadine is based on nterference with the viral protein, M2 (an ion channel), the protein needed for the viral particle to become uncoated once it is taken inside the cell by endocytosis [61]. Also, oseltamivir carboxylate mechanism implies a selective inhibition of influenza virus neuraminidase enzymes, which are glycoproteins found on the virion surface, very important for viral entry into uninfected cells, for the release of recently formed virus particles from infected cells, and for the further spread of the infectious virus in the body [62,63].

Hepatitis viruses have been the subject of intense study in the last years, with a special attention on therapy. As mentioned in the first section, hepatitis treatment depends upon the type of hepatitis, therefore different antivirals are considered and summarized in detail in references [64,65,66,67,68,69]. Currently, interferons (IFNs) α, β, and γ have antiviral activity, the first two being produced by nearly all cells as response to viral infections, while the third is restricted to T-lymphocytes and NK cells. IFN-induced proteins include 2′-5′-oligoadenylate [2-5(A)] synthetase and a protein kinase, either of which can inhibit protein synthesis in the presence of double-stranded RNA. The 2-5(A) synthetase produces adenylate oligomers that activate a latent cellular endoribonuclease (RNase L) to cleave both cellular and viral single-stranded RNAs.

Antiretroviral therapy—ART—refers to the treatment with HIV medicines. According to the last updated list approved by Food and Drug Administration (FDA) these drugs can be classified as can be seen in Figure 2 [70,71]. In the following paragraph the authors describe the pharmacological mechanism of the medicines for HIV treatment that were included/encapsulated/incorporated into nanomaterials. The representative nucleoside reverse transcriptase inhibitor (NRTI) zidovudine (AZT) is phosphorylated intracellular by kinases specific to AZT 5′-triphosphate, a metabolite responsible for termination in elongation of proviral DNA because it is incorporated by reverse transcriptase into nascent DNA but lacks a 3′-hydroxyl group. Resistance to AZT is associated with mutations at reverse transcriptase codons 41, 44, 67, 70, 210, 215, and 219 [72]. Also, lamivudine (LAM), another NRTI agent, enters cells by passive diffusion, and then is converted to the monophosphate by deoxycytidine kinase, and undergoes further phosphorylation by deoxycytidine monophosphate kinase and nucleoside diphosphate kinase to yield lamivudine 5′-triphosphate, which is the active anabolite [73]. Tenofovir disoproxil is a derivative of adenosine 5′-monophosphate lacking a complete ribose ring, and it is the only nucleotide analogue currently marketed for the treatment of HIV infection, being active against HIV-1, HIV-2 and HBV. After a rapid hydrolysis, tenofovir is formed, being then phosphorylated by cellular kinases to its active metabolite, tenofovir diphosphate which is a competitive inhibitor of viral reverse transcriptases and is incorporated into HIV DNA, causing chain termination [74,75].

Non-nucleoside reverse transcriptase inhibitors (NNRTIs) include a variety of chemical substrates that bind to a hydrophobic pocket in the p66 subunit of the HIV-1 reverse transcriptase and induce a conformational change in the 3D structure of the enzyme that greatly reduces its activity, and thus they act as non-competitive inhibitors. Unlike nucleoside and nucleotide reverse transcriptase inhibitors, these compounds do not require intracellular phosphorylation to attain activity [76]. Also, the binding site for NNRTIs is virus-strain-specific and the approved agents are active against HIV-1 but not HIV-2 or other retroviruses and should not be used to treat HIV-2 infection.

HIV protease inhibitors (PIs) are peptide-like chemicals that competitively inhibit the action of the virus aspartyl protease (a homodimer consisting of two 99-amino acid monomers). The preferred cleavage site for this enzyme is the N-terminal side of proline residues, especially between phenylalanine and proline. These drugs prevent proteolytic cleavage of HIV gag and pol precursor polypeptides that include essential structural (p17, p24, p9, and p7) and enzymatic (reverse transcriptase, protease, and integrase) components of the virus, preventing the metamorphosis of HIV virus particles into their mature infectious form [77,78]. Only SQV, indinavir, ATV, RTV, NFV and lopinavir are currently employed in nanotechnology research.

There are two drugs available in the entry inhibitors class: enfuvirtide—ENF and maraviroc—MCV, with different mechanisms of action, both incorporated into nanoparticles as specified in Table 2. ENF inhibits fusion of the viral and cell membranes mediated by gp41 and CD4 interactions, while MCV is a chemokine receptor antagonist and binds to the host cell CCR5 receptor to block binding of viral gp120. As such, MCV is the only approved antiretroviral drug that targets a host protein [79,80,81].

Raltegravir (RAL), the first approved HIV integrase inhibitor, has potent activity against both HIV-1 and HIV-2, and also retains activity against viruses that have become resistant to antiretroviral agents of other classes because of its unique mechanism of action [81,82,83,84]. RAL was encapsulated into a polymeric PLGA nano-formulation and gold nanoparticles (see Table 2).

## 3. Biological Barriers Security System

The first line of defence [85] that any substance encounters is the biological barriers penetration into the organism. The “security” system includes physiological barriers, such as blood-brain barrier (BBB), epithelium, stratum corneum, air-blood lung barrier [86], reproductive system barrier, etc. all of which control the extracellular and intracellular access and trafficking of foreign substances such as bacteria, viruses, fungi, and chemicals [87] but also provide selective access to “suitable candidates” such as nutrition and/or therapy molecules.

It is well established that more than one mechanism may be involved in intracellular drug delivery. The mechanisms involved in nano-based intracellular drug delivery include passive diffusion of free drug, non-specific phagocytosis of the nanocarrier, nanocarrier uptake by pinocytosis, and receptor-mediated endocytosis [88]. In this section, we will discuss in particular how to overcome biological barriers such as mucus, skin, cell membrane and BBB in antiviral therapy.

### 3.1. Mucus

The gastrointestinal tract, respiratory system, the urogenital cavities, eyes and mouth, are all covered with mucosal membranes. The highly adhesive mucus acts as protective layer as well as for lubrication purposes. Although large molecules cannot pass, small ones along with viruses can easily penetrate. These are also the reasons that drug delivery is so challenging. Mucus contains 95% of water along with mucin fibres, lipids, salts, cholesterol and proteins. It is continuously produced but the thickness, pH and amount differ by its position.

Two strategies have to be followed for passing through mucus, mainly depending how fast the turnover is: fast mucosal penetration or highly adhesive particles (slow turnover) to increase the drug’s residence on the targeted mucosa.

Mucoadhesion can be the resultant of interactions like hydrophobic, hydrogen bonding, ionic bonding or van der Waals ones. Other possible attractive interactions can be covalent bond formation between catechols, maleimides, thiols, and acrylates with domains of mucin glycoproteins rich in cysteine. Chitosan, alginate, pectin or cellulose polymers are mostly used for achieving adhesion on mucosa. Furthermore, it is known that thiolation of the mentioned polymers develops high mucoadhesive properties.

Mucopenetration is the second strategy found to permeate the mucus layers by two potential mechanisms: active strategy characterized by the interaction with the mucus and chemically shifting the features of the mucus or their own structures and passive strategy that uses hydrophilicity enhancers to penetrate the mucus [89].

The classical HSV therapy includes daily dosing of orally administered ACV [90] that is effective in most cases, and of course problematic in other cases, for example, in long-term use of ACV patients report resistance against the drug followed by renal injury [91,92]. Another issue produced by standard HSV treatment in this case for the topical form of trifluridine (TFT) and ganciclovir (ACV analog) gels, is denoted by low retention time on vaginal and corneal mucus followed by multiple doses up to 10 times [93].

The nanotechnology field offers a great deal of drug delivery modalities in order to overpass the biological barriers, deliver efficiently the incorporated active principle in a controlled and targeted manner, reduce circulating drug levels and attenuate the renal damage. A recent review points out the nanogels based on the above-mentioned materials capabilities as an adequate example to pass through these types of biological barriers [94]. For example, the synthesized nano-drug delivery micelles based on chitosan-*g*-oligo (NiPAam) copolymers stabilised by ionotropic crosslinking by Raskin et al. [95], gave good results for delivering antiretroviral drugs (EFV) through mucosa. More studies on how ACV penetrates different barriers can be found in Table 2 and detailed in Section 5.

It is important to mention a “special barrier” namely the ocular mucus that changes completely in 5 to 8 min, making the drug absorption unfavourable. In addition, the eye is protected by blood-anterior chamber and blood-retinal barriers. In case of drug administration, a combination of penetration and adhesion of the substances is necessary. Polymers like phosphotyrosine could be the solution. It was demonstrated that if intestinal alkaline phosphatase is present, polymers can manifest a zeta potential change, thus causing their immobilization after penetration. Another kind of construct for combining adhesion and penetration is through thiolated systems with mucolytic enzymes, pH dependent. Therefore, at acid pH, the absence of disulphide bonds formation with cysteine-rich domains in mucins does not manifest mucosal adhesion unless they are near the epithelium [94].

### 3.2. Skin

Skin, as the largest organ of our body, protects us from microorganisms and chemicals, regulates our body temperature and maintains hydroelectrolytic balance. The two layers of the skin are the epidermis and the dermis. The first one is avascular and is composed of stratified, keratinized squamous epithelium, in four layers from bottom up: basale, spinosum, granulosum and corneum. Thick skin (palms and soles) has a fifth layer (under the most superficial corneum) called lucidum. The dermis consists of two layers (reticular and the more superficial papillary) of connective tissue of elastin and collagenous fibres and has in its component blood and lymphatic vessels network, nerves, touch receptors (Meissner corpuscles), adipocytes, phagocytes, hair follicles and sweat glands [96].

Topical, through skin drug delivery, has a local effect, requiring less drug for the targeted outcome. Transdermal therapy results in fewer side effects with no need of regular treatment but systemic distribution of the drug. Both methods of treatment have a common blockage, stratum corneum. To pass through this barrier, different approaches were developed based mostly on disrupting this structure chemically with substances like surfactants, alcohols, esters, amines, terpenes, alkanes phospholipids, or mechanically by using ultrasounds, micro needling, magnetophoresis, iontophoresis, electroporation or lasers. Excessive use, though, can damage the skin.

Analysing the literature data, we can suggest that there are different processes and mechanisms that govern the penetration of small/large molecules through skin barrier. According to Schneider et al. [97] review there are two general pathways for skin absorption: through skin appendages or through the stratum corneum and the underlying layers. The lipophilic statum corneum medium determines the first mechanism of skin penetration, namely absorption of lipophilic compounds [98]. The three transport routes of substances across stratum corneum can be classified in transcellular, intercellular and trans-appendageal pathways as defined by Liang et al. [98]. More examples are available but the conclusion is the same: “the full understanding of the penetration or absorption processes is still under evaluation” due to the challenges associated with delivering complex burdens through the skin barrie [99].

**Figure 3:**
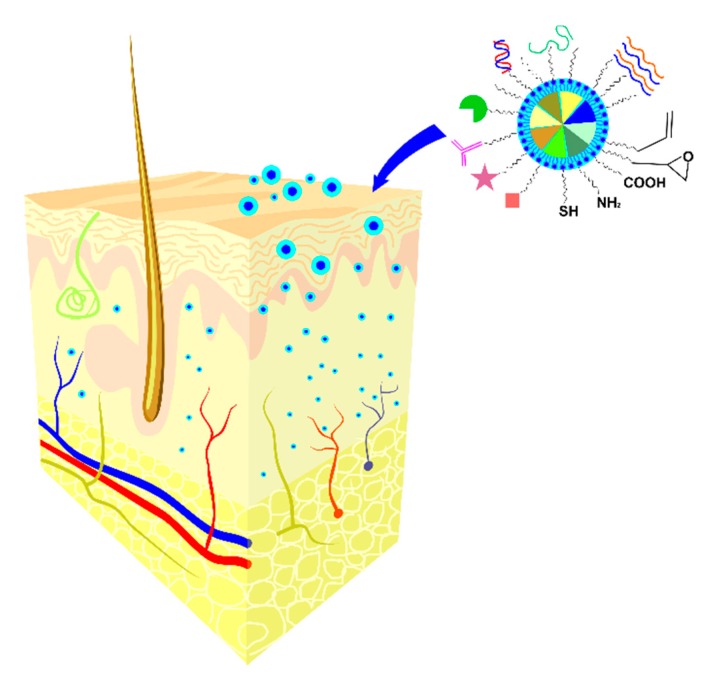
Schematic illustration of nano-scale carrier systems and their interactions with the dermal barrier.

As specified in Section 2, the current antiviral therapy for HSV infection includes topical formulations of ACV that is unable permeate stratum corneum and target the virus site at the basal epidermis due to its polarity and solubility, leading to poor clinical efficacy due to delayed antiviral activity and sub-inhibitory concentrations [100]. Nanotechnology strategies [97] seem to facilitate the “admission fees” due to the rationally design and innovative functionalities of the synthesized nano-platforms as presented in Figure 3.

One more problem in dermal drug passing is related to inflammation skin pathology. The barrier is changed, and the drug resides much less on the targeted site because of fast penetration. The thermoresponsive drug delivery nanogels used in this purpose have encouraging results for overcoming the above-mentioned problems. pH sensitive nanogels can also be utilised for controlled medicinal release.

Thermoresponsive nanogels can be controlled through irradiation with infrared lamp. Another way of skin penetration is the hair follicle. The stratum corneum is less intact in the lower infundibulum, so the nanoparticle’s (NPs) passage is dependent on size and not on composition [94].

### 3.3. Cell Membrane

The cell membrane separates the content of a cell from the exterior surroundings. Besides the standard protection around the cell, the cell membrane controls what substances go in and out. The composition is based on a bilayer of phospholipids, internally hydrophobic (tails) and externally hydrophilic (heads) with different proteins and cholesterol between them. The membrane is permeable selectively, permitting only some materials to pass through its lipid layer by active (through protein pumps or vesicles) or passive (diffusion) processes of transportation. Water passes the membrane through a process called osmosis, which occurs when an imbalance of solutes appears outside and inside the cell [101].

In most of the cases, hydrophobic and small molecules can pass through diffusion. Nanoscale drug delivery systems depend upon an active mechanism (endocytosis). Over this process, the cell unit takes in ions, solid particles and molecules. There are studies that prove that positive charged nanogels can bind the membrane of the cell (negative charge) through electrostatic intercommunication. More than that, receptor-mediated endocytosis can provide a mechanism through which selectively attracted cell groups are targeted. In addition, hydrophobic nano-platforms can grow the adhesion to the membrane and the amount of drug entered in the cell [94].

### 3.4. Blood-Brain Barrier

The BBB is a highly selective semipermeable structure composed of five parts: the basement membrane, the astrocytes, the immune cells, the pericytes and an endothelial cell layer of capillaries. The area between basement membrane and the neurons is called Virchow-Robin space. In this region there is interstitial fluid in which reside microglia. All the above components are called neurovascular unit. The kinetics of this unit is crucial to the role of BBB and its states of illness. The capillaries of BBB compose a layer of squamous epithelial cells that fold to form a circular vessel. These cells are linked with strong connections for blocking entrance or exit of materials through central nervous system. Protein transportation facilitates the selective flow of molecules through vessel lumens, essential biomolecules being in higher concentrations, like glucose and at the same time eliminating toxins.

The BBB is a physical and metabolic “obstacle”, physiologically important and active, which survey blood-brain traffic and control it, restricting the paracellular diffusion between the endothelial cells (microvessels) and the efflux pumps activity that quickly expel back into the capillary lumen a wide variety of xenobiotics. BBB integrity and function is critically influenced by what is now referred to as “the extended neurovascular unit” that incorporates not only microvascular endothelial cells and adjacent pericytes, astrocytes and neurons, but also neighbouring smooth muscle cells and microglia in the brain, and blood cells in the capillary lumen such as polymorphonuclear cells, lymphocytes and monocytes [102].

Transport at the BBB level is assured by numerous transport mechanisms that provides to the brain the necessary nutrients and also protects from the toxic xenobiotics. The main transport mechanisms are represented by free diffusion of small lipophilic substances or by active transport (carrier mediated, receptor mediated and active efflux transport).

Active efflux transport is assured by two major types of transporters that extrude metabolic waste, xenobiotics and a large number of drugs from the brain back into the blood. The first superfamily of BBB efflux transporters is the solute carrier proteins (SLC) superfamily, being represented at the level of BBB by SLC22 and SLCO (SLC21) efflux transporters. The second is the ATP-binding cassette (ABC) efflux transporter family represented by permeability glycoprotein (P-gp), breast cancer resistance protein (BCRP) and the multidrug resistance associated proteins (MRPs) [103,104,105].

Based on their localisation, the ABC efflux transporters prevent lipophilic and amphiphilic environmental toxic compounds or drugs, including anti-inflammatory, immunosuppressive, anti-infectious, antineoplastic drugs, some antiepileptic, antidepressant and psychotropic agents, and drug conjugates by an energy-dependent, unidirectional direct transport mechanism, from entering specific substrates [106]. The BBB’s efflux machinery does an excellent job of recognizing xenobiotics, but a poor job on distinguishing between toxicants and therapeutic drugs, creating an important obstacle to treatment of brain cancer, epilepsy and neuro AIDS [107].

The penetration of the BBB for drug delivery, although challenging, captivates the interest of numerous researchers in antiviral therapy since the mechanisms by which for example HSV-1 penetrates the CNS remain unclear. The most likely routes include retrograde transport via the olfactory or trigeminal nerve fibres, occasionally leading to herpes simplex encephalitis (HSE) caused by HSV-1 [108].

Another studied virus that is involved in encephalitis and BBB disruption is HIV, known to cause severe neurological disorders and leading to HIV-related encephalitis [109] since BBB is impermeable to 98% of antiretroviral drugs [110]. The possible mechanism responsible for BBB disruption in HIV-1 encephalitis is considered a “Trojan horse” mechanism, where HIV infects specific T-lymphocytes and circulating monocytes, then entering to CNS through BBB gaps and followed by inflammatory reactions [111], but, in the last years nanotechnology has been intensely explored and several experimental attempts have been carried out in order to enhance the BBB permeability toward antiretroviral drugs, briefly described below based on the nano-based formulation composition, since it is well known that the size and surface functionalization influence transport properties within tissues:

- polymeric polybutylcyanoacrylate (PBCA) nanoparticles with two incorporeated antiretroviral drugs (AZT and lamivudine) showed a 8–20 and 10–18 fold increase in BBB permeation, by three possible mechanisms as presented by the authors: prolonged interaction interval between drug-loaded nanoparticles and brain-microvascular endothelial cells elevated the concentration gradient between blood and the brain, Polysorbate 80 covering on the periphery of nanoparticles was able to be absorbed and degraded nanoparticles improved drug absorption [112];
- spherical transferrin coated-PEGylated albumin nanoparticles encapsulating AZT prepared by ultra-emulsification method using chemical cross-linking by glutaraldehyde gained an access across the BBB through the transferrin receptor mediated endocytosis on the membrane [113];
- transferrin-conjugated quantum rod nanoparticles conjugated with saquinavir crossed an in vitro BBB model by exploiting a receptor-mediated transport [114];
- magnetic liposomal nanoformulations of azidothymidine 5′-triphosphate (the active form of azidothymidine) migrate across BBB in vitro, either directly or by a monocyte-mediated transport, under the influence of an external magnetic field [115];
- novel nanodrug consisting of an iron oxide nanoparticle coated with PMA amphiphilic polymer and functionalized with the antiretroviral peptide enfuvirtide crossed the BBB by a passive diffusion, probably mediated by the absorption of the amphiphilic coating on the cell membrane [116].

As briefly presented the preliminary results are more than encouraging. Certainly, future investigations on the mechanisms about BBB disruption are needed along with novel, innovative, safe and efficacious therapeutic approaches.

## 4. Nanotechnology: How Does It Face the Antiviral Therapy?

Up to now we have concluded that current antiviral therapy has not yet achieved the ideal shape and efficiency and also that the complex biological barriers are major obstacles, but can we critically say that nanotechnology could be the identified solution?

Search engine queries on “nanotechnology” generate more than 114,000 items on specialized platforms (Science Direct, for example) that represents potential and challenges in different fields from biosensors and industry-related applications up to nanomedicine and biomaterials. When the search keywords are “nanotechnology as antiviral therapy” the same engine only returns 1404 results starting from 1997.

It is a given fact that nanotechnology is defined as the application of materials on the nanometer scale. According to the literature data results, namomaterials designed with different shapes and morphologies display numerous advantages for use in antiviral therapy, namely: nanometric size that permits drug delivery through impermeable barriers [88], large surface area to volume ratios for large drug payloads incorporation [117] and improved efficacy, surface modification and/or backbone functionalization versatility that facilitates cellular membranes passage [118] or enhancing stability and bioavailability [119], virucidal activity against a series of viruses (HIV, HSV, HBV, etc.) due to biomimetic properties [120], increased specificity, improved antiviral delivery and controlled drug release to the target [121] through engineered moieties, decrease the emergence of drug resistance, personalized therapy possibility, protection of the drugs and low adverse drug side effects mainly due to the composition. 

**Figure 4:**
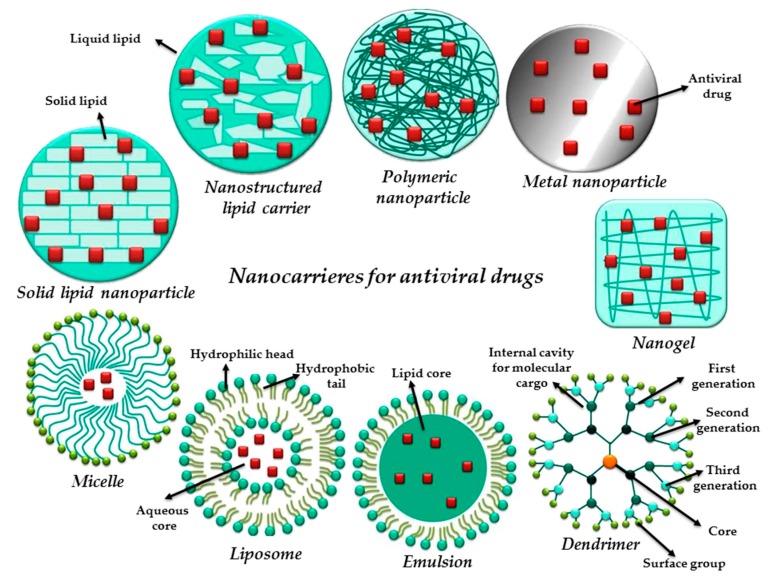
Nanocarriers developed for antiviral therapy.

The mechanisms of nanomaterial-mediated drug delivery are determined by the chemistry, the architecture and the specific properties of each nanosystem (as presented in the schematic representation in Figure 4).

The design of new drug delivery systems for the antiviral therapy is focused on manipulating these features that are relevant in viral diseases where high drug doses are compulsory, implies high costs and the patient is depended on the administration protocol.

Lembo and Cavalli [18] present the current status up to 2010 in the nanoparticulate delivery systems in antiviral therapy area, highlighting their perspective on the challenges that must be tackled before the nanotechnology can be translated into clinical use as safe and effective antiviral formulations. Therefore, the nanoparticulate antiviral systems synthesised up to 2010 consisted mainly of micelles, polymeric NPs, solid lipid NPs (SLNs), nanostructured lipid carriers (NLCs), liposomes, nanocapsules, vesicles, dendrimers, nanogels, cyclodextrin-based systems and emulsions.

In 2016, Liu and Chen [122] summarized in a review paper an interesting perspective of nanotechnology use in HIV/AIDS vaccine development. Their overview underline the potential of various nanomaterials and nano-architectures to be used as HIV vaccine carriers or adjuvants due to proven capabilities to improve delivery, permeability, stability, solubility and pharmacokinetics of traditional HIV vaccine approaches. The authors exhibit also the desired features of nano-carriers and adjuvants with high benefits-cost ratio.

In 2017, Milovanovic et al. [123] outlined, beside, the virus replication cycle and mechanism of actions of antiviral agents, an overview of particulate carriers for drug delivery. The review summarized several classes of the mostly considered carriers namely liposomes, micelles, microspheres, dendrimers and NPs synthesized as alternative supports for antiviral therapy. Table 2 highlights part of their summary and described based on virus classification in Section 5.

In 2019, Cao and Woodrow [124] reviewed the nanotechnology solutions used to eradicate HIV reservoirs and also the gene delivery and immunotherapy nanocarriers used in cancer with potential in HIV treatment. In Section 4. Nanocarriers for eradicating HIV reservoirs” the authors focused mainly on nanocarriers incorporating combination therapeutics developed in order to boost drug effectiveness and minimize toxicity. Several examples are presented in Table 2 and described in Section 5.

Recently, Arca-Lafuente et al. [163] overviewed nanotechnology-based systems as reliable alternative diagnostic tools for HCV infectious disease. Even if our review does not cover screening, it is important to mention that new diagnostic methods are required in order to overcome current drawbacks of HCV under-diagnosed infection as highlighted in the above-mentioned review. The nanotechnology-based tools described in the review seem to fulfil the necessary features for HCV elimination.

With the aim of developing new personalized diagnostic tools, Farzin et al. [164] summarized current strategies and under-development tools for early diagnosis of HIV. Their review combines the use of nanomaterials such as carbon nanostructures, nanoclusters, quantum dots, metallic and metal oxide NPs as advanced structures for HIV detection with possible biosensing strategies targeting to offer innovative outlooks for developing intelligent, sensitive and specific nano-objects for in situ and real-time detection of HIV.

## 5. Current Overview of Nanotechnology Use in Antiviral Therapy: Virus Related

In this section the authors point out the suitability of nanomaterials (recent data) for antiviral therapy, highlighting the enhanced features pursued to overcome the identified issues as related above.

### 5.1. Nanomaterials Designed for Non-Retroviral Antiviral Agents

#### 5.1.1. Nano-Based Antiviral Agents against Herpes Viruses

Donalisio et al. [155] have reported the preparation by a modified nano-emulsion method of chitosan nanospheres (NS) loaded with 8.5% ACV as a topical formulation against both HSV-1 and HSV-2 herpes virus strains. The main component, chitosan, a natural polycationic polysaccharide, was selected as a material for ACV release, due to its distinctive properties: hydrophilic character, in situ gelling, mucoadhesion, permeation enhancing, in addition to a low cytotoxicity, biocompatibility and bioresorbability features [165]. The obtained gel formulation based on ACV-loaded NS proved an enhanced ability to penetrate porcine skin to about 55% (at 24 h) greater than the commercial cream product (10%). IC_50_ values against HSV-1 and HSV-2 were also determined on Vero cell cultures infected with above-mentioned strains, displaying significant reduced values of 0.012 µM and 0.100 µM, respectively, when using the NS formulation as compare to 0.156 µM and 1.608 µM for free acyclovir. This nano-technological approach attests the higher efficacy of the described formulation and with promising expectations for further preclinical and clinical experiments.

Yadavalli et al. [133] have explored the potential of highly porous activated carbon (HPAC) particles as a model for restricting HSV-1 and HSV-2 from entering target cells. They have considered this material due to the charcoal surface-active that could provide antiviral effects through virion sequestration approach. Furthermore, ACV molecules adsorbed or encapsulated inside the HPAC pores revealed sustained drug release acting in a synergistic manner to obtain an enhanced therapeutic effect. The HPAC compound prove d a 40 to 60% reduction in HSV-1 and HSV-2 entry for concentrations as low as 1 mg/mL. The IC_50_ value of HPAC corresponding to HSV-1 and HSV-2 infection in prophylactic administration was found of 0.8 and 1 mg/mL, respectively, significantly lower than clinically accepted TC50 value (half maximal toxicity concentration) of HPAC. Following the promising outcomes from in vitro tests, further determinations of antiviral efficacy on in vivo studies using a murine model of ocular (HSV-1) and genital (HSV-2) infection were performed. As a result, the ACV loaded HPAC acts by capturing the virus and releasing the encapsulated drug, hindering inflammation and immune cell infiltration in targeted tissue. The strong antiviral activity of this product was assigned to the charged surface of its pores which may interact with the cell’s surface, stimulating an active exchange of ions (Na^+^, K^+^, Ca^+^, Cl^−^, and OH^−^), when sustained or slow release of ACV has been acquired. Moreover, these particles exhibited both prophylactic and therapeutic effects against HSV-1/HSV-2 cells, unlike the free drug that did not demonstrate a prophylactically antiviral response.

#### 5.1.2. Nanomaterials with Antiviral Intrinsic Activity

##### Gold and Silver NPs Using Seaweed *Sargassum wightii* with Anti-Herpetic Activity

Biogenic gold (Au) and silver (Ag) NPs were prepared using the seaweed *Sargassum wightii* (*Sw*) and investigated for their antiviral activity against HSV-1 and HSV-2 strains [166]. The NPs synthesis resided in an eco-friendly method, previously described in the literature [167], replacing the use of different reducing agents. The obtained NPs, *Sw*-Au and *Sw*-Ag, were evaluated concerning both cytotoxic and antiviral effect, using MTT and CPE (cytopathic effect) assays on Vero cells. The results showed that cell viability ranged from 93% to 85% when the concentration ranged between 2.5 and 25 µL per sample in *Sw*-Au, and from 97% to 84.58% for concentrations of 2.5 and 1 µL per sample in *Sw*-Ag. The antiviral assay have shown a 70% decrease of CPE on both HSV-1 and HSV-2 when Vero cells were treated with 10 and 25 µL *Sw*-Au, whereas *Sw*-Ag exhibit similar reduction of CPE at a concentration of only 2.5 µL per sample. Higher concentrations of *Sw*-Ag are not accepted due to an increased cytotoxic effect. The authors claimed that the obtained results are in agreement with other published research and they inferred that functionalized metallic NPs act as antiviral agents by blocking the virus attachments and cell access, depending on particle size.

##### Broad-Spectrum Antiviral NPs

Cagno et al. [168] conducted a research study concerning broad-spectrum antiviral products, which usually mimic heparan sulfate proteoglycans (HSPG), as well-preserved target of “viral attachment ligands” (VALs). The antiviral effect relies on the binding mechanism of the nanoparticles to the virus surface, thus preventing virus-cell attachment. In most cases, the reversibility of these bonds is reported [169], so that by increasing the dilution viral inhibition is lost, causing those compounds not to be considered antiviral drugs. The aforementioned authors have designed antiviral nanoparticles of virucidal effect based on long and flexible linkers simulating HSPG, leading to irreversible viral deformation. Of the synthesized compounds, the most notable virucidal effect was found in the AuNPs coated with a 2:1 mixture of decanesulfonic acid (MUS) and 1-octanethiol (OT). MUS allows a multivalent binding [170] as a consequence of its structure comprising a long hydrophobic chain, sulfonic acid terminated. The enhanced activity of MUS:OT-NPs was assigned to the new construct using MUS linker that caused local distortions and then a global virus deformation, leading to irreversible loss of infectivity. The MUS:OT-NPs exhibited efficient virucidal effect against HSV-1 and HSV-2, human papilloma virus (HPV-16), respiratory syncytial virus (RSV), dengue and lenti virus. The in vivo testing on Balb/c mice infected with RSV reveals the efficacy of MUS:OT-NPs treatment that prevented the pulmonary dissemination of the infection. These results are in agreement with previous published data [171], which assessed the relationship between the surface structure of nano-objects and their ability to cross cell membranes. Both in vivo and in vitro tests on cell cultures have proven the lack of toxicity of MUS:OT-NPs.

##### Lipid Nanoemulsions Encapsulating Coumestrol as Topical Treatment of Herpes Simplex

Coumestrol is an isoflavonoid-like compound having the ability to inhibit the replication of HSV-1 (both acyclovir sensitive and resistant strains) and also some strains of HSV-2 [172]. Argenta et al. [173] have designed a formulation in an effort to obtain a topical product for coumestrol delivery at the level of mucosa. In this approach, the bioactive compound was entrapped by fluid or rigid phospholipid nanoemulsions (dioleylphosphocholine, DOPC and distearoylphosphocholine, DSPC, respectively) dispersed in a hydroxyethylcellulose gel. The effectiveness of the proposed antiviral agents was tested regarding permeation and retention ability on intact and damaged porcine esophageal mucosae and for antiherpes activity on cell culture assays using Vero and GMK AH1 cell lines. The greatest performance of both coumestrol-loaded nanoemulsions NE-COU/DOPC and the same product thickened with hydroxyethylcellulose, HNE-COU/DOPC, as compared to those based on DSPC, relies largely on the physico-chemical properties of the nanoemulsion. The positively charged nanoemulsion showing highest values of ζ-potential may interact with negatively charged surface of mucosa membrane, with beneficial consequences relating to transmucosal delivery of coumestrol [174]. The length of phospholipids alkyl chain, the number of unsaturations, the lipophilic/hydrophilic balance of the active principle also contributed to the global effect, so that the fluid-state of hydrocarbon chain induced by DOPC explained the interaction between the oil-water interface and mucosa, increasing coumestrol permeation and retention. The low IC_50_ values proved an enhanced antiviral activity against HSV-1 and HSV-2 after coumestrol formulation using nanoemulsions based on DOPC, which could be considered for advanced studies in order to be introduced in therapy.

### 5.2. Nanomaterials Designed for Antiretroviral Drug Delivery

The huge socio-economic impact of HIV, as mentioned in Section 1, determined a continuously increased trend of studies related with finding an almost perfect treatment. Since the seven classes of antiretroviral drugs defined by FDA contain a large number of active principles, plenty of studies regarding their incorporation in different types of nanosystems can be found in the literature. Several examples are presented below.

#### 5.2.1. NRTIs and NNRTIs

RTIs classes, NRTIs and NNRTIs, include some of the drugs often used in HIV treatment plans. Recently, Grande et al. [175] published a complex review on RTIs nanosystems for controlled drug delivery and our review complements part of the data presented in this article, emphasizing the penetration of biological barriers in vitro or in vivo by nanosystems containing RTIs.

AZT, a high bioavailable drug, has serious side effects, the most common being bone marrow suppression, toxicity for some organs, neutropenia and anaemia. Specific target drug delivery using different nanosystems is a promising solution [140]. ATZ has been incorporated in hybrid NPs based on alginate and stearic acid-poly ethylene glycol. C6 glioma, neuro brain and Hela cells have been used to study the cellular uptake and the cytotoxicity of the NPs in vitro. The results proved that these nanosystems are nontoxic and have significant brain cellular uptake, suggesting that they can be used for more complex internalization in brain cells studies [176]. In another study, sol-oil chemistry has been used to prepare small NPs lactoferrin loaded with AZT (50–60 nm in size), stable in biological simulated fluids (gastric and intestinal). Antiviral activity of NPs has been analysed using SupT1 Cells infected with HIV-193IN101 virus and the results suggested that the encapsulation of AZT in lactoferrin does not influence the drug activity. The NPs loaded with AZT and the drug alone have been orally administrated to Wistar rats of both genders, the performed assays (bone marrow micronucleus, histopathological and biochemical analysis) showing that AZT loaded in NPs is more efficient and less toxic, compared with the soluble form [140].

Lamivudine—LAM, a water-soluble drug with two main drawbacks: its half-life is only 2 h and has a deficient bioavailability, especially in paediatric patients (68%) [177]. LAM has been included in NPs based on poly(ε-caprolactone)—PCL [178,179], poly lactic-*co*-glycolic acid—PLGA [141], chitosan and sodium alginate [180], Eudragit E100 [177]. The physico-chemical characterization of the obtained NPs has shown an adequate size of the NPs and a good stability. In vitro drug release tests indicate that NPs can support the drug delivery for 24 h, indicating a less frequent administration [179,180]. Sneba et al. [177] have reported a more complex study, where LAM-polymeric non-cytotoxic NPs have been included in films for drug delivery through the buccal mucosa barrier. Four mucoadhesive polymers: polyvinyl alcohol—PVA, polyvinyl pyrrolidone—PVP, sodium carboxymethylcellulose—SCM, hydroxypropyl methylcellulose—HPMC have been used to prepare the films. Moreover, Ozturk et al. [141], obtained PLGA NPs loaded with LAM and proved that are physicochemical stable and slowly released the drug, a great property attributed to ester end-group of PLGA. Because these NPs were intended for oral administration, the authors evaluated the gastrointestinal stability of the NPs in vitro, using different fluids of biological interest with pH in the range 1.2–7.4 phosphate buffer solution, intestinal fluid phosphate buffer solution, physiological serum and distilled water; the tests have been developed at 37 °C for 24 h. The results indicated that PLGA NPs are promising intestinal targeted drug delivery system for LMA, being stable in tested media.

In clinical practice, LAM is frequently administrated together with AZT, therefore this combination of drugs is being studied for target delivery using different types of NPs. Sankar et al. [142] used PLGA, methylmethacrylate-sulfopropylmethacrylate—MMA-SPM, poly lactic acid—PLA, and poly methyl methacrylate—PMMA to prepare different types of NPs by emulsion polymerization, as drug delivery nanosystems for ATZ (52%) and LMA (58%). In vivo acute toxicity has been studied in mice; the results proving the fact that the drug doses loaded in the NPs are not toxic. ATZ-LAM PLGA NPs seemed to be the most promising nanosystems.

Efavirenz—EFV, one of the most used NNRTIs in clinical practice, is a poorly water-soluble drug, and the incorporation in different drug delivery nanosystems being a solution for this drawback. Patel et al. [156] obtained nanosuspensions—NS based on povidone polymer- polyvinylpyrrolidone (PVP) K30, poloxamers steric stabilizer (188 and 407) and an anionic electrostatic stabilizer (sodium lauryl sulphate, a steric stabilizer—SLS). Compared with the drug alone, an important improvement of saturation solubility has been noticed for the NS with EFV. The incorneporation of EFV in NS increased the absorption of the drug in rat intestine in situ, and very important the oral bioavailability in the studies on Albino rabbits. Lactoferin used to prepare NPs loaded with ATZ [140], as described above, has been used also to encapsulate EFV [146], based on the same preparation technique: sol-oil chemistry. Compared with free EFV, the encapsulation of the drug in NPs showed a reduced toxicity to peripheral blood mononuclear cells, Jurkat T cells and B16-F10 cells, an increased anti-HIV-1 activity and improved oral bioavailability and pharmacokinetic profile in studies on rats.

#### 5.2.2. PIs

ATV. Low brain permeability and antiretroviral drug resistance are two of the most important disadvantages of ATV. This PIs drug has been encapsulated in SLNs and studied as nanosystems for brain delivery using hCMEC/D3 as a blood-brain barrier in vitro model, HCMEC/D3 being human brain microvessel endothelial cell line. Average ATV had an important increase regarding the cellular uptake once delivered through average nanosystems (around 167 nm) [159].

SQV, another anti-HIV PIs, has been incorporated in poly(ethylene oxide)—modified poly (ε-caprolactone) (PEO-PCL) NPs [143] by a solvent displacement process. Human monocyte/macrophage (Mo/Mac) cell line—THP-1 has been used for in vitro cellular uptake assay. The drug has been successively released intracellular and a meaningful uptake of the SQV-PEO-PCL has been noticed.

NFV, used in HIV-1 and HIV-2 treatment as PI, is a promising drug that can be used also for other grave medical disorders like cancer [181]. Some studies have showed the ability of different types of NPs loaded with NFV to activate latent HIV and to restrict viral spread in vitro. Kovochich et al. [160] showed that lipid NPs–LNPs incorporated with bryostatin-2, a protein kinase C activator (LNP-Bry), can be loaded with NFV (LNP-Bry-NFV), and proved the above mentioned abilities on J-Lat Full Length Cells (10.6). Tang et al. [144] have prepared NPs based on poly(lactic-*co*-glycolic acid)-polyethylene glycol diblock copolymers and anti-CD45RO antibody conjugated with suberoylanilide hydroxamic acid (SAHA) and NFV and tested theirs in vitro properties on ACH-2 cells. More complex studies have been performed by Venkatesh et al. [145] PLGA NPs loaded with NFV have significantly enhanced the oral bioavailability of the drug studied in vivo in New Zealand rabbits, a reduced frequency of dosing being needed in this case.

The literature data reports also several studies where combinations of PIs drugs have been incorporated in nanosystems and pre-clinically evaluated. Duan et al. [161] have included separately ATV and DRV in LNPs, but only ATV-LNPs proved to form stable drug-lipid concentrations. Based on these results, the authors have developed LNPs containing ATV and RTV and also LNPs containing ATV + RTV + tenofovir (TFV—an HIV NRTIs), the last ones being prepared in a large volume for a preliminary primate pharmacokinetic study. After LNPs subcutaneously administration, the three drugs have been detected in plasma for seven days.

#### 5.2.3. Fusion Inhibitors, Entry Inhibitors and Integrase Inhibitors

ENF is a fusion inhibitor that is incapable to cross the cerebrospinal fluid. Fiandra et al. [116] proved that by including it into a nanosystem composed from magnetic nanoparticles (MNP) synthesized by solvothermal decomposition in organic solvent followed by fluorescent labelled PMA coating could solve ENF drawback. In vitro model (co-colture of RBMVECs and astrocytes) and in vivo model (Balb/c mice) studies proved that nanoconjugated ENF could penetrate BBB.

MCV, an entry inhibitor acting as a CCR5 co-receptor antagonist, has been also included in some nanosystems in order to increase its oral bioavailability. Solid drug nanoparticles—SDNs, containing 70 wt % MVC and 30% some polymer/surfactant excipients have been prepared using the emulsion-template freeze drying technique. Monolayers of Caco-2 have been used as a human gut in vitro model in order to study the absorption behaviour of MVC SDNs and in vivo oral pharmacokinetics of the MVC solid drug nanoparticles (SDNs) has been analysed on a rat model. Both studies indicated an advanced permeability of the MCV NPs (based on PVA and sodium 1,4-bis(2-ethylhexoxy)-1,4-dioxobutane-2-sulfonate (AOT) excipients) correlated with the normal drug [148].

Bowman et al. [182] proved that small organic monovalent molecules conjugated to AuNPs acts as fusion inhibitors in vitro, while Vijayakumar et al. [183] proved that AuNPs alone acts as entry inhibitors. Moreover, integrase inhibitor, RAL, has been functionalized with a thiol group in order to link Au-NPs. In vitro cellular uptake has been tested on macrophages, human brain microendothelial cells and primary peripheral blood mononuclear cells, the results suggesting that RAL—pMBA—Au-NPs penetrate the cells and also can exhibit antiviral activity. In vivo studies performed by injection of RAL—pMBA—Au-NPs in female adult BALB mice tail proved that the studied NPs could cross the BBB [184].

## 6. Progress in Nanomedicine: Antiviral Nanotherapeutics Approved or under Evaluation

Nanomedicine represents a fast-revolutionizing field that faces rapidly and constantly progress assessed by the numerous nanodrugs that have entered clinical practice and also by even more being investigated in clinical trials. Table 3 presents the approved antiviral nanomedicines, from which half are vaccines.

According to Singh et al. [185] review from 2017 and also to available information on the respective websites there are still several nanomedicines under evaluation, namely:

- Fluquit (STP 702) from Sirnaomics Inc. currently under preclinical evaluation, a polymer-based nanotherapeutic that incorporates siRNA and targeting the H5N1 (avian flu), H1N1 (swine flu) influenza, and newly emerging H7N9; and cervisil (STP909), a nanobased drug candidate, which incorporates siRNA for the treatment of HPV16 and HPV18;
- DermaVir from Genetic Immunity, a synthetic pathogen-like nanomedicine that incorporates single plasmid DNA expressing 15 HIV antigens that assemble to HIV-like particles; DermaVir vaccine completed Phase I/II randomized, placebo-controlled, dose-finding, double-blinded, multicenter study to assess the safety, tolerability and immune response in HIV-1-infected adults who are currently receiving anti-HIV treatment (number NCT00270205) [194];
- Doravirine (MK-1439), from Merck, a novel, next generation NNRTI described as solid drug nanoparticle formulation tested for HIV; currently doravirine completed the pharmacokinetic trial of the bioavailability of four MK-1439 nano formulations in healthy adults (number NCT02549040) [195];
- Lipid nanoparticles of ARB-001467 TKM-HBV containing three RNAi therapeutics for HBV genome targeting from Arbutus Biopharma; in 2018 the company completed the phase 2a, single blind, randomized, placebo controlled, study evaluating the safety, anti-viral activity, and pharmacokinetics (PK) following multiple doses of intravenous ARB-001467 (number NCT02631096) [196].

## 7. Authors’ Perspective to Design Next Generation of Nano-Based Antivirals for Clinical Translation

As discussed above, nanotechnology started to be a critical player in the antiviral therapy. As mentioned by Ross et al. [99], nanotechnology frees the current therapy payloads in terms of delivery across biological complex barriers, and could resolve the low bioavailability drawback as already stated in Section 3.

Nanomaterials impart many physical, chemical and biological advantages [18,197] such as: (1) small particle size in order to facilitate drug delivery through biological barriers, (2) large surface area to volume ratios to ensure large drug payloads, (3) tunable surface charge to facilitate cellular entry across the negatively charged cellular membrane, (4) biomimetic properties which result in intrinsic antiviral assets, (5) ability to anchor targeting moieties to increase specificity to desired cell types, tissue or other compartments, (6) improved solubility and pharmacokinetic and/or pharmacodynamics properties translated in longer time to allow greater accumulation, controlled and sustained release, (7) enhanced efficiency gained either by drug molecules entrapment to protect them from physiologically hostile media, or by using surface conjugation to target drugs to specific tissues, (8) reduced toxicity and (9) multifunctionality by combining several beneficial features in a stable construct, designed to simultaneously stimulate the replication of latent virus and deliver an antiviral to the activated cell [198].

Several limitations were also acknowledged such as: (1) degradation, for example nanoparticles are degraded in the gut following oral administration, or fail to penetrate the mucus barrier and are thus minimally absorbed [199], (2) undesired interactions with biological molecules that leads to opsonization, uptake by macrophages and reduced plasma half-life [200], (3) non-specifically absorption that may induce apoptosis and disrupt cell membrane and adverse immunological responses [201], (4) large dimension for renal clearance therefore cannot be degraded within the body, and are accumulated, leading to toxicity [202], and (5) scaling up issues and high costs.

In our perspective, the “ideal” nanocarrier for efficient antiviral delivery must take into considerations several key factors namely:

- Clinical outcome, since patients need safe, effective, targeted, available and affordable therapy, as they are our inspiration;
- From the clinical perspective, the future antiviral candidates should improve the efficacy of the fused/encapsulated drug, reduce the intake frequency and time, restrict adverse side effects and reduce therapy costs;
- Design consideration for the nanoplatforms that will allow targeted delivery of the drugs in sustained released manner and improves efficacy, safety and patient convenience; therefore, from a chemist point of view, hybrid nanosystems can gather all the necessary features in terms of composition, shape and size by overcoming limitations of individual systems and offers greater advantages. Starting with the composition, the chosen materials should be biodegradable, biocompatible, and non-toxic, for example polymers are very attractive since they offer the possibility for chemical modifications over the surface or backbone. In addition to these advantages, the second component from the hybrid architecture (in the shape of potential liposomes) should offer besides advanced barrier penetration, higher encapsulation efficiency for the intended drug, which in combination with the polymeric piece will be able to modulate the release kinetics, the stability and prolong drug release. When thinking about the shape, we have in mind targeting capabilities as impact. As we already know, the shape is linked with size and surface charge and density, therefore a complex puzzle that must be solved. The surface charge and density should be carefully chosen during the nanoplatforms design through the surface modification possibility. The ideal candidate here from our perspective is PEG due to its versatility to exhibit various charges, shapes and sizes but also to enhance tolerability, reduce clearance, and lengthen circulation time. The size influences the biodistribution and the uptake rate therefore the “nominee” has to be in the submicron size range, recommended to be under 200 nm.

Taking into consideration the performance indicators of nanomedicine, we claim that the development a personalized nanomedicine is possible via a synergistically approach. Since the development of “best” viral carriers involves a multidisciplinary team, virologists should be directly implicated in the development, offering specialized support on the following matters: identification of differentially expressed moieties virus cells for targeted delivery, elucidation of the type of desired targeted and the response from the host cells to nanodelivery platforms.

Therefore, multidisciplinary research-oriented efforts have to be related also to system biology by exploring machine learning for process optimization and pharmacology in order to introduce best appropriate combination of therapeutic agents.

In 2018, an interdisciplinary team of virologists and biochemists, which developed low-cost and “cell-friendly” nanogels that can efficiently prevent viral infections, addressed these challenges [203]. Here, the flexible, nontoxic and broad-spectrum nanogels based on dendritic polyglycerol sulfate mimic cellular surface receptors where several viral families bind. The designed nanogels can multivalently interact with viral glycoproteins, shield virus surfaces, and efficiently block infection since they act as robust inhibitors for these viruses.

When thinking about the translation into the clinical practice, the nano-based future antiviral therapy must follow a specific flow-chart, starting with the optimization and scale-up practices according to the good manufacturing practice, the elaboration of suitable regulatory guidelines and finishing with the development of cost-effective and high quality formulations available worldwide. Taken into consideration all these enhanced features, the road to clinical practice still has many addressed issues in order to provide effective and safe antiviral nano-formulations to patients.

## 8. Conclusions

Treating or improving treatment success rate for viral diseases are fundamental responsibilities. The established potential and boosted progress of nanotechnology in antiviral therapy development generates great expectations for new therapeutic innovative strategies for attacking or eradicating viral disorders. At present, studies explored numerous and diverse nano-platforms including nanoparticles, liposomes, micelles, with different compositions, size, with single or combined entrapped drugs that may serve as potential antiviral drug delivery transporters. These nano-based systems have exhibited versatile features to improve the identified current therapy drawback. However, the clinical use of a nano-based antiviral formulation to date based on our knowledge has turned out just a few approved or under clinical trials nanoformulations, mainly vaccines, despite more than 22 years of constant efforts. It is expected in the upcoming years that part of this “success preclinical story” to be scaled-up, translated and applied for better outcome, convenience and access for patients.

## Figures and Tables

**Table 1:** Common viral infections.

Viral Infection	Viruses	References
Common cold	rhinoviruses, parainfluenza viruses, respiratory syncytial viruses coronaviruses, Influenza viruses, adenoviruses, enteroviruses, metapneumovirus, unknown	[21]
Eye infections	herpes simplex virus, adenovirus, cytomegalovirus	[22,23,24]
Encephalitis or meningitis	as JC virus, measles, LCM virus, arbovirus, rabies	[25]
Pneumonia	influenza virus (A and B), parainfluenza virus, respiratory syncytial virus, adenovirus, SARS coronavirus	[26]
Cardiovascular and pancreas disease	coxsackie virus;	[27,28,29]
Hepatitis	hepatitis viruses types A, B, C, D, E	[30,31,32,33]
Skin infections	varicella-zoster virus, human herpesvirus 6, smallpox, molluscum contagiosum, human papillomavirus, parvovirus B19, rubella, measles, coxsackie A virus	[34,35,36,37,38]
Gastroenteritis	adenoviruses, rotaviruses, noroviruses, astroviruses, coronaviruses	[39,40,41]
Sexually transmitted diseases	herpes simplex type 2, human papillomavirus HIV	[42]

**Table 2:** Nano-delivery systems developed for antiviral drugs.

Nanoplatform Type	Nanoplatform Characteristics (Size, Morphology, Toxicity etc.)	Drug	Virus Type	REF
**Liposomes**				
- Reverse phase evaporation	GCV mixed with PC/CH/NaDC dissolved in chloroform/diethyl; Spherical liposomes; Liposome sizes of 210 ± 17 nm, ζ-potential—52.4 mV; polydisperse;	GCV	HSV	[125]
- rHDL	rHDL-nosiheptide complex with a diameter < 30 nm;	Nosiheptide	HBV	[126]
	rDHL-ACV palmitate complex size of 33.5 nm, around 10 times smaller than ACV-lipososmes;	ACV		[127]
- cationic	Viral gene expression reduce by 65–75% in liver after 2 days of administration at mice;	siRNA	HCV	[128]
- immunoliposomes	Viral secretion reduced by 81% and free viral particles neutralized in vitro;	HIV gp 120 Folding inhibitor	HIV	[129,130,131]
	In vivo resistance to infection has been enhanced;	anti-CCR5 siRNA		
	Immunoliposomes diameter with average between 100 and 120 nm; really useful to deliver high concentrations of indinavir;	Indinavir		
- pegylated	In house synthesized pis; reduced toxicity and increased adherence in vitro;	PIs		[132]
**Nanoparticles**				
- HPAC	HPAC (different concentrations)—non-cytotoxic for human epithelial cells (corneal, vaginal), HeLa cells, foreskin fibroblasts: cell viability >75%; 99% drug loading efficiency;	ACV	HSV	[133]
- PLGA	Three GCV pro-drugs have been separately loaded on PLGA NPs; Uniform, spherical and smooth surface nps; Particle size between 116 and 143 nm; ζ-potential between −13.8 and −15 mv; Non-cytotoxic PLGA-nps (24 h and 48 h contact of three different NPs concentrations with HCEC cell);	GCV	HSV-1	[134]
- Se	Uniformly spherical Se@AM; Se@AM size of 70 nm, compared with SeNPs size which is 200 nm; Se@AM—more stable than SeNPs; Se@AM—superior antivirial effect on kidney cells treated with H1N1 and less citotoxicity (79.26% viability) than SeNPs (58.8%) or free AM (53.23%);	AM	H1N1	[135]
	Uniformly spherical Se@OTV Se@OTV size of 100 nm; Se@OTV—superior antivirial effect on kidney cells treated with H1N1 and less citotoxicity (93% viability) than SeNPs (60%) or free OTV (53%);	OTV		[136]
- Ag	Monodisperse and uniformly spherical particles; Ag@AM size of 2 nm; highly stable NPs for more than 28 days; NPs loaded with AM on their surface less cytotoxic (90%) than free AM (56%) or AgNPs (65%);	AM		[137]
- PEG-PLGA	Uniformly spherical shape; Size of NPs loaded with: diphyllin—178 nm and bafilomycin—197 nm; Superior biocompatibility and antiviral activity for the drugs loded on NPs than the free drugs;	Diphyllin and Bafilomycin		[138]
- Human serum albumin + copolymers of maleic anhydride/alkyl vinyl ethers of oligo (ethylene glycol)	Mean size of NPs in the range of 100–300 nm; NPs surface with targeting moieties able to interact with liver cells receptors;	INFs-α	HIV	[139]
- Tf-Albumin-PEG	Spherical particles; NPs size between 114 and 124 nm; NPs surface had a negative charge;	AZT		[113]
- Lactoferrin	Spherical AZT-lactoferrin particles with the diameter in the range of 50–60 nm; The drug was intact after the preparation process; AZT encapsulated in lactorferrin NPs is more efficient and less genotoxic (Wistar rats) compared to free AZT;			[140]
- PLGA NPs	Polydisperse particles loaded with LAM with the size between 221 and 250 nm; ζ-potential between −4.64 and −3.65 mv; The molecular interaction between LAM and the polymer confirmed by FTIR and DSC; Slow degradation of NPs in simulated intestinal fluid PBS;	LAM		[141]
- Hybrid NPs (PLGA, MMA-SPM, PLA and PMMA)	PLGA NPs size between 58–224 nm; MMA-SPM NPs size between 91–823 nm; Almost spherical NPs; Non-toxic NPs (male mice);	LAM+AZT		[142]
- PEO-PCL	Spherical PEO-PCL NPs with smooth surface; PEO-PCL size around 200 nm, PEO-PCL size around 270 nm; SQV was encapsulated into NPs;	SQV		[143]
- PLGA-PEG	Spherical shape; Size of the NPs: 125 nm for those loaded with SAHA and NFV; 118 nm for those loaded with NFV; 119 nm for those loaded with SAHA; Low cytotoxicity effects of NPs loaded with the drugs (tested on ACH-*2* cells);	SAHA NFV		[144]
- PLGA	Spherical and smooth surface NFV NPs with mean size of 185 nm; an almost narrow distribution; ζ-potential of 28 mV;	NFV		[145]
- Lactoferrin	NPs mean size of 45–60 nm, hydrodynamic radius of 103 nm, ζ-potential around −23 mV; polydisperse NPs; Chemically stable NPs proved by FTIR and DSC;	EFV		[146]
- Folic acid-conjugated-P407	NPs based on folic acid conjugated with P407 with inclusion of ATV and RTV significantly decreased the amount of HIV produced by cells in mice;	ATV+RTV		[147]
- PMA coated MNP	Uniformly spherical NPs conjugated with ENF with size of 35.2 nm and ζ-potential around −29 mV; Non-toxic in vitro and in vivo NPs conjugated with ENF;	ENF		[116]
- PVA-AOT	Particles with diameters between 658 and 823 nm; ζ-potential between −12.8 and −25.3 mV;	MCV		[148]
- pMBA-Au NPs	NPs with diameter of 1.8 nm; Non-toxic in vitro NPs;	RAL		[130]
- PLGA	NPs average size of 138.3 nm and ζ-potential around −13.7 mV; Non-cytotoxic NPs loaded with drugs compared to free NPs;	EFV+Lopinavir+RTV		[149]
- PLGA+Pluronic F127	Well-defined polydisperse NPs with average size of 220 nm and −19.2 mV ζ-potential; No adverse events and toxicity in a 14 days pharmacokinetic study on mice;	TAF+EVG		[150]
- Lactoferrin	Average drug loaded particles size of 67 nm; Insignificant in vitro toxicity to red blood cells; Improved bioaviability of the three drugs;	AZT+EFV+LAM		[151]
**Dendrimers**				
- PG	Non-toxic peptide–PG conjugates in vitro; Antiviral effect in vitro;	Peptides	IAV	[152]
- Alginate-PEG	Dendritic structure confirmed by TEM; Hydrodynamic diameter in the range of 601–782 nm and ζ-potential in the range of −45.8 and −65 mv; Great biocompatibility proved by the values of cell viability: between 88% and 98% (neuro cells, Hela cells, glioma cells);	AZT	HIV	[118]
**Nanorods**				
- PVP-PEG coated with Ag	The presence of AM on the surface of Ag nanorods confirmed by FTIR; The nanorods loaded with AM of 540 nm diameter. Acceptable viability of cells (hela, huvecs, dendritic cells, macrophages) after 72 h contact with three different concentrations of AM nanorods;	AM	HIV	[153]
- Tf-conjugated QRs	QR-Tf-SQV hydrodynamic diameter is 130–140 nm; QR-Tf with different SQV concentrations non-cytotoxic after 6–48 h of contact with BMVECs;	SQV		[154]
**Nanospheres**				
- Cs	Spherical NS with an almost smooth surface; average diameter around 200 nm, polydisperse NS, ζ-potential around 40 mV; ACV encapsulation efficiency 86% Satisfactory Vero cell viability after contact with NS;	ACV	HSV	[155]
**Micelles**				
- Cs-*g*-oligo(NiPAam)	Copolymers self-assembled into multimicellar aggregates with hydrodynamic diameter between 330 and 436 nm and ζ-potential between +7 and +22.8 mV; good mucoadhesion and cytocompatibility properties;	EFV	HIV	[95]
**Nanosuspensions**				
- zirconium oxide beads stabilized with PVP, poloxamers and SLS	Mean particele size around 320 nm, ζ-potential −32.8 mV; EFV bioavailability improved after incorporation in nanosuspensions (in vivo, rabits);	EFV	HIV	[156]
**Nanoemulsions**				
- Mucoadhesive NEs	Based on triacetin-oil, tween 20-surfactant, transcutol P-cosurfactant; 23–200 nm spherical particles; Nontoxic and noniritant nanoplatforms (New Zealand albino rabbit);	GCV	HSV	[157]
**SLNs**				
- Borneol	Microemulsion-based method; Particle size between 113 and 142 nm; ζ-potential between −15.1 and −18.3 mV; polydisperse particles;	GCV	CMV	[158]
- Stearic acid + Pluronic F68)	Spherical SLNs with a mean diameter of 167 nm and ζ-potential around −18 mV; SLNs loaded with AZT can successfully deliver the drug in vitro to human brain endothelial cells;	AZT	HIV	[159]
**Lipid nanoparticles**				
- bryostatin-2	The lipid nanoparticles can stimulate latent HIV and can inhibit virus spread in vitro;	NFV	HIV	[160]
- PEG and phospholipids	Size of LNPs loaded with drug/drugs between 33 and 68 nm and incorporation efficiency between 88% and 96%;	ATV+RTV		[161]
		ATV+RTV+TFV		
- DSPC+MPEG+DSPE	Particle diameter between 52 and 68 nm; In vivo anti-HIV LNPS do not exhibit local reactions and animal platelet counts are within normal limits.	Liponavir+RTV+TFV		[162]

**Table 3:** Examples of approved nanoplatforms for drug delivery by the FDA, EMA and other organizations (updated table, after Singh et al. [185]).

Name	Company/Approval Year/Country/Organization	Nanoplatform. Benefits	Virus	Route of Administration	REF
Epaxal^®^	Crucell (former Berna Biotech Ltd.); 1994 Switzerland	Virosomes (around 150 nm *spherical liposomal vesicles*)—intrinsic adjuvant properties; reduced toxicity and superior tolerability;	HAV	Intramuscular vaccine	[186,187]
Inflexal^®^ V	Crucell (former Berna Biotech Ltd.); 1997 Switzerland	Virosomes (around 150 nm *spherical liposomal vesicles*)—biodegradable and biocompatible adjuvant systems; unwanted side effects; superior immune response;	Influenza	Intramuscular vaccine	[187,188]
PegIntron^®^	Schering Corporation, 2001, U.S., FDA	PEG-interferon alfa-2b (polymeric NPs) —31.000 Daltons molecules; superior protein stability;	HCV	Subcutaneous	[189]
Pegasys^®^	Genentech, 2002, U.S. FDA	PEG-interferon alfa-2a (polymeric NPs)—31.000 Daltons molecules; superior protein stability;	HBV, HCV	Subcutaneous	[190,191]
Influvac^®^ Plus	BGP Pharma ULC, 2005, Canada	Virosome vaccine	Influenza	Intramuscular vaccine	[192]
VivaGel^®^ BV	Starpharma, Australia; Mundipharma, Europe, 2019	Dendrimer (astodrimer sodium—SPL7013) incorporated in a water-based vaginal gel, acting as a targeting antiviral biofilm.	HIV, HSV	Topically Applied (Vaginal gel)	[193]

## Abbreviations

**AIDS**: acquired immunodeficiency syndrome; **ACV**: acyclovir; **Ag**: silver; **AM**: amantadine; **ART**: antiretroviral therapy; **ATV**: atazanavir; **AZT**: zidovudine; **BMVECs**: brain microvascular endothelial cells; **Au**: gold; **CCK-8**: Cell Counting Kit-8; **CH:** cholesterol; **CMV**: cytomegalovirus; **Cs**: chitosan; **DAA:** direct-acting antiviral; **DSC**: differential scanning calorimetry; **DLS**: dynamic light-scattering; **DNA**: Deoxyribonucleic acid; **DsDNA**: double stranded DNA; **DRV**: darunavir; **DSPC**: 1,2-Distearoyl-sn-glycero-3-phosphocholine; **EFV**: efavirenz; **EVG**: elvitegravir; **FDA**: Food and Drug Administration; **FTIR**: Fourier-transform infrared spectroscopy; **GCV**: gangiclovir; **GMP**: good manufacturing practice; **AuNPs**: gold nanoparticles; **HBV**: hepatitis B virus; **HCV**: hepatitis C virus; **HSV**: herpes Simplex; **HIV**: human immunodeficiency virus; **HPAC**: highly porous activated carbon; **HPMC**: hydroxypropyl methylcellulose; **IAV**: influenza A virus; **IFNs**: interferons; **LAM**: lamivudine; **LNPs**: lipid nanoparticles; **MNPs**: magnetic nanoparticles; **MPEG**: *N*-(carbonylmethoxypolyethyleneglycol-2000); **DSPE**: 1,2-distearoyl-sn-glycero-3-phosphoethanolamine, sodium salt; **MTT**: [3-(4,5-Dimethylthiazol-2-yl)-2,5-Diphenyltetrazolium Bromide]; **MTS**: [3-(4,5-dimethylthiazol-2-yl)-5-(3-carboxymethoxyphenyl)-2-(4-sulfophenyl)-2H-tetrazolium]; **NaDC**: sodium deoxycholate; **NEs**: nanoemuslions; **NiPAam**: *N*-isopropylacrylamide; **NLCs**: nanostructured lipid carriers; **NPs**: nanoparticles; **NRTIs**: nucleoside and nucleotide reverse transcriptase inhibitors; **NNRTIs**: non-nucleoside reverse transcriptase inhibitors; **NS**: nanospheres; **OTV**: oseltamivir; **PBS**: phosphate buffer solution; **PC**: phosphatidylcholine; **PCL**: poly(ε-caprolactone); **PEG**: poly(ethylene glycol); **PEO-PCL**: poly(ethylene oxide)-modified poly(ε-caprolactone); **PG**: polyglycerol; **PLGA**: poly lactic-*co*-glycolic acid; **PIs**: protease inhibitors; **pMBA**: p-mercaptobenzoic acid; **PVA**: polyvinyl alcohol; **PVP**: poly(vinylpyrrolidone); **QRs**: quantum rods; **RAL**: raltegravir; **REF**: reference; **rHDL**: recombinant high density lipoproteins; **RNA**: Ribonucleic acid; **RTIs**: Reverse transcriptase inhibitors; **RTV**: ritonavir; **SCM**: sodium carboxymethylcellulose; **SLNs**: solid lipid nanoparticles; **SLS**: sodium lauryl sulphate; **SAHA**: suberoylanilide hydroxamic acid; **SEM**: scanning electron microscopy; **SeNPs**: selenium nanoparticles; **Se@AM**: selenium nanoparticles with amantadine; **siRNA**: small interfering RNA; **ssDNA**: single stranded DNA; **ssRNA**: single stranded RNA; **TAF**: tenofovir alafenamide; **TEM**: transmission electron microscopy; **Tf**: transferrin; **TFV**: tenofovir; **UNAIDS**: The Joint United Nations Programme on HIV/AIDS; **VZV**: varicella-zosterian virus; **ζ-potential**: zeta potential.
